# Error Properties of Argos Satellite Telemetry Locations Using Least Squares and Kalman Filtering

**DOI:** 10.1371/journal.pone.0063051

**Published:** 2013-05-17

**Authors:** Janice D. Boyd, Donald J. Brightsmith

**Affiliations:** 1 The Parrot Fund, College Station, Texas, United States of America; 2 Schubot Exotic Bird Health Center of the Department of Veterinary Pathobiology, Texas A&M University, College Station, Texas, United States of America; University of Alberta, Canada

## Abstract

Study of animal movements is key for understanding their ecology and facilitating their conservation. The Argos satellite system is a valuable tool for tracking species which move long distances, inhabit remote areas, and are otherwise difficult to track with traditional VHF telemetry and are not suitable for GPS systems. Previous research has raised doubts about the magnitude of position errors quoted by the satellite service provider CLS. In addition, no peer-reviewed publications have evaluated the usefulness of the CLS supplied error ellipses nor the accuracy of the new Kalman filtering (KF) processing method. Using transmitters hung from towers and trees in southeastern Peru, we show the Argos error ellipses generally contain <25% of the true locations and therefore do not adequately describe the true location errors. We also find that KF processing does not significantly increase location accuracy. The errors for both LS and KF processing methods were found to be lognormally distributed, which has important repercussions for error calculation, statistical analysis, and data interpretation. In brief, “good” positions (location codes 3, 2, 1, A) are accurate to about 2 km, while 0 and B locations are accurate to about 5–10 km. However, due to the lognormal distribution of the errors, larger outliers are to be expected in all location codes and need to be accounted for in the user’s data processing. We evaluate five different empirical error estimates and find that 68% lognormal error ellipses provided the most useful error estimates. Longitude errors are larger than latitude errors by a factor of 2 to 3, supporting the use of elliptical error ellipses. Numerous studies over the past 15 years have also found fault with the CLS-claimed error estimates yet CLS has failed to correct their misleading information. We hope this will be reversed in the near future.

## Introduction

Documenting animal movements is key for understanding species’ home ranges, migration patterns, resource tracking, and is vital for developing realistic conservation plans. Remote tracking of animals began in the late 1950’s with VHF radio telemetry and this technique is still frequently used to track terrestrial animals over relatively short distances [1]. However, tracking of wide-ranging animals and intercontinental migrants was not possible until the 1970s with the development of 5–11 kg Argos system PTTs (platform terminal transmitters) for tracking large mammals [2]. The second generation of smaller, lighter satellite transmitters appeared in the mid to late 1980s and weighed as little as 110–150 g [3]. By the late 1990s PTTS as light as 30 g became available [3], with further size and weight reductions limited by current battery and solar cell technology. For some telemetry applications the newer GPS (Global Positioning System) has replaced VHF and Argos. An important advantage of both Argos and GPS is that, unlike short-range VHF telemetry, the satellites are placed in orbits that allow positions to be obtained from every location on earth, allowing studies of wide ranging and migratory animals in inaccessible regions both terrestrial and marine. However, the use of GPS is limited by the need to download stored positions or the need for a data relay system to transmit positions to a distant user – often via VHF or Argos. Present GPS-based systems with data relay are generally not light enough for deployment on animals weighing <1000 g (i.e., maximum ∼ 30 g, using the maximum 3% of body weight rule of thumb) for more than several days or a few weeks– largely because of power limitations [4], [5], [6], [7], [8], [9], [10].

Quantifying location error is a key component of all telemetry studies as it allows users to realistically analyze and interpret their data (e.g., [11], [12], [13]). CLS, the French entity that operates the Argos system (hereafter referred to as “Argos”) provides theoretical estimates of the errors of its positions and sends users estimates of these errors with each computed location. Unfortunately, Argos does not make clear if these error estimates refer to the precision (reproducibility) or the accuracy (deviation from true location) of the positions. Given that most users probably find accuracy estimates most useful they likely assume that this is what “error estimates” refer to.

The primary error descriptor for Argos locations is the location code or location class (LC) [14]. The LC is based upon the estimated error in the positions and number of messages the satellite receives from the PTT. There are seven different location codes, 3, 2, 1, 0, A, B, and Z, with 3 presumed to have the smallest error, B the greatest, and Z considered an “invalid location.” By default, users receive only positions with LCs 3, 2, and 1. To receive positions with LCs 0, A, B, and Z users must request “Service Location Plus/Auxiliary Location Processing” from Argos User Services [14]. Position errors are assumed by Argos to follow a bivariate normal distribution [15]. Each location code is assigned a general one-dimensional error value (Table 1) assumed to include 68% of positions with that LC: 250 m for LC 3, 500 m for LC 2, 1500 m for LC 1 and >1500 m for LC 0. Argos does not provide estimated errors for LC A or B.

**Table 1:** Classification and 1-dimensional accuracy of location classes as provided by Argos.

	Estimated error (68^th^ percentile)		Messages required per satellite pass (as per Argos)	
Location Code, LC	Least Squares	Kalman Filter	Least Squares	Kalman Filter
3	<250 m		4 messages or more	
2	250 m<<500 m		4 messages or more	
1	500 m<<1500 m		4 messages or more	
0*	>1500 m		4 messages or more	
A*	No accuracy estimation		Unbounded accuracy estimation	
B*	No accuracy estimation		2 messages	
Z*	Invalid location		3 messages	1 or 2 messages

* By default, users receive positions with LC 1, 2, and 3 only. To receive LC 0, A, B, and Z positions (indicated by *), users must request “Service Location Plus/Auxiliary Location Processing” from Argos User Services. Reproduced from Argos Users’ Manual Section 3.4.

The satellites carrying Argos equipment are polar orbiting, and as a result the true error around calculated positions is better represented by 2-dimensional ellipses rather than 1-dimensional circles [14]. From the covariance matrix of the messages received by the satellite, Argos derives an error ellipse with major and minor axes “a” and “b” plus the ellipse orientation [15]. Argos encourages use of the two-dimensional error ellipses for more precise quantifying of the location error, although these parameters are only available as Diagnostic Data from ArgosWeb and in table form from ArgosDirect if requested from User Services [14].

The realism of Argos-quoted location errors has been the subject of substantial investigation (e.g., [16], [17], [18], [20], [21], [22], [23]). Uniformly, these papers have reported that the 1-dimensional error estimates provided by Argos greatly underestimate the observed errors using a variety of different PTTs at different sites. However we have found no evaluations of the realism of the Argos error ellipses and resulting 2-dimensional errors.

Several researchers have also noted that a lognormal distribution appears to better describe the 1-dimensional error than a normal distribution [19], [21] although the implications of this have not been explored. Other papers have used a t-distribution after noting the non-Gaussian nature of the error distributions [22], [24], but it appears that no statistical tests were run to see how well the t-distribution actually fit the data or compared the fit to other possible distributions. Vincent et al. [23] mentioned that after filtering and smoothing, data grouped by location code gave some datasets that could be fit by a normal distribution.

At the present time, Argos provides two algorithms for calculating locations: least squares (LS) and Kalman filtering (KF). Users must select only one method for receiving near-real-time data; to obtain both the user must request and pay for post-experiment reprocessing. All previous studies of Argos position errors have been done with the LS method, which has remained unchanged since 2007 [14] but modified a number of times prior to that. The KF method – a technique very different from LS [14] – was made available as a user option in March 2011. Once implemented operationally, Argos recommended users switch to KF processing because it “introduces significant improvements in the number of positions and their accuracy, especially for applications where just a few messages are received per satellite pass or for platforms operating in difficult transmission conditions” [14]. However, this claim has not been independently verified.

In this study we test the accuracy of the locations provided by Argos and compare errors (defined by us as the difference between true location and Argos location) from LS and KF processing techniques with two collars developed for use on large macaws (*Ara* ssp) at our research site in southeastern Peru. In an effort to provide location error estimates useful for interpreting Argos-generated animal movement data, we also calculated and evaluated empirical error estimates based on our data. We test the following hypotheses: 1) the 1-dimensional error distributions for both LS and KF processing methods are lognormally distributed, 2) data processed using KF provide more locations of higher accuracy than data processed using LS, 3) Argos-provided error estimates characterize the actual accuracy for both processing methods, and 4) Argos-provided error ellipses around computed locations provide a useful way of characterizing the position accuracy in 2 dimensions.

## Materials and Methods

The study was conducted at the Tambopata Research Center in the Department of Madre de Dios in southeastern Peru (13°8′S, 69°36′W) under permit number 030-2009-SERNANP-DGANO-JEF. The center is on the border of the Tambopata National Reserve (274,690 ha) and the Bahuaja-Sonene National Park (1,091,416 ha) in the Department of Madre de Dios. The center lies in the tropical moist forest life zone near the boundary with subtropical wet forest at 250 m elevation and receives about 3200 mm of rain per year [25], [26]. The site is surrounded by a matrix of mature floodplain forest, successional floodplain forest, *Mauritia flexuosa* (Arecaceae) palm swamp, and upland forest [27].

Data were collected in 2009–2010 with two low power (250 mW) Argos transmitters (PTTs) designed specifically for us for use on large macaws (*Ara* spp). Two telemetry companies provided the PTTs: Telonics (model TAV-2627) and North Star Science and Technology (custom). Both units were of similar design with a narrow metal band wrapping around the bird’s neck. Attached to the neckband was an electronics canister hanging under the bird’s beak against the chest at roughly crop level. An antenna about 21 cm long extended from the end of the canister up past the neck. The units weighed 32 g (North Star) and 37 g (Telonics), with components weighing about 3.5 g for electronics, 9 g for battery, and 20+ g for damage resistant housing, neckband and antenna. The Telonics unit was slightly heavier because the electronics housing was filled with potting material. For programming the on-off duty cycles, the North Star unit had an internal timer and the Telonics unit a clock; we found the latter easier to use.

We hung the two collars from tall rainforest trees and towers near the Tambopata Research Center to estimate expected position errors for our macaw movement studies. We realize that our position errors are likely conservative, as accuracy on free-ranging macaws will likely be worse than under these static conditions. One collar (North Star) transmitted from late March 2009 through mid-February 2010 (331 days); the other (Telonics) from early February 2009 through late May 2010 (477 days). Transmission took place on a varying schedule of every three or four days for four to six consecutive hours during each transmission period. There was no obvious schedule impact on the number or quality of resulting locations. The positions from both the LS processing method and the newer KF processing method were compared with the known positions of the trees and towers.

For monitoring purposes, LS locations were received directly from Argos for the duration of the study. Our data from before the KF implementation in 2011 were reprocessed at our request by Argos. Our analysis was performed using the LS and KF data sent in the reprocessed data package.

Analysis was done using Excel and standard or custom-written scripts in MATLAB^©^. We computed errors in the Argos calculated locations as distances and directions from the known positions of the transmitters with all directions calculated in compass convention as clockwise from north. To obtain the most accurate distance measures possible on the approximately spherical Earth, we calculated distances and azimuths using Vincenty’s algorithm [28] as available from the MATLAB Central file exchange as VDIST [29] and VDISTINV [30].

Data were grouped into 14 separate subsets for processing according to location code (N = 7) and processing method (N = 2). Given that Argos error may be influenced by variation in PTTs, we used a Kolmogorov – Smirnov test to test the hypothesis that the errors from the two PTTs came from the same distribution. This was done by independently comparing the error magnitude from the two transmitters for all 14 location code and processing method combinations.

Probability plots and Lilliefor’s tests were generated for the datasets to test goodness of fit of the presumed candidate distributions – normal and lognormal (using logarithm to the base e). In all statistical tests we used a significance level (alpha) of 0.05 unless otherwise noted. For the 14 position error datasets we computed statistics appropriate for normally distributed data (mean, standard deviation, 68^th^ percent confidence intervals, 68^th^ percentiles) and statistics appropriate for lognormally distributed data (geometric mean, multiplicative standard deviation, 68% confidence intervals and 68^th^ percentiles) from the relationships in Table 2 for comparison with the Table 1 values from Argos. The geometric mean (m*) and the multiplicative standard deviation (s*) were calculated following the recommendations and formulas in Limpert et al. [31].

**Table 2:** Relationship between parameters characterizing normal (y) and lognormal (x) distributions.

Property	Normal Distribution, y = log(x)	Lognormal Distribution, x = exp(y)
Central limit theorem	Additive effects	Multiplicative effects
Distribution shape	Symmetrical	Skewed
Definitions: Characterizing parameters		
Measure of central tendency	m, arithmetic mean of y	m*, geometric mean of x = exp(m)
Standard deviation	s, standard deviation or additive standard deviation	s*, multiplicative standard deviation = exp(s)
Measure of dispersion	Coefficient of variation = s/m	s*
Definition: Confidence interval or two sided confidence limits	m ± z·s:	[m*/(s*)^z^, m*·(s*)^z^ ]
68^th^ percent confidence interval	m ±1·s	[m*/(s*), m*·s* ]
90^th^ percent confidence interval	m ±1.645·s	[m*/(s*)^1.645^, m*·(s*)^1.645^ ]
Definition: Percentile or upper one-sided confidence bound	m+z·s	m* (s*)^z^
50^th^ percentile	m (mean)	m*
68^th^ percentile	m +0.4677·s	m* · (s*)^0.4677^
90^th^ percentile	m +1.282 · s	m* · (s*)^1.282^

By “z” is meant the appropriate value of the standard normal variate. Percentage and percentile calculations are exact for a population and are approximate for a sample from a population. Confidence limits give the expected two-sided limits enclosing the specified percentage of the observations. The confidence bound gives the expected percentage of observations lying at or below the indicated value. Adapted from [31].

We computed the numbers of LS and KF positions for each location code and noted the number of positions that changed location code after reprocessing with KF. To examine if Kalman processing improved position accuracy of individual points over least squares processing, we created time- and position-matched datasets consisting of all LS positions grouped by location code and all the matching KF results, discarding the new KF locations that had not been computed under LS. Log transforming the datasets gave approximately normal datasets with which we performed analysis of variance and Tukey multiple comparison tests.

To examine the relationship between the computed compass bearing of the error (the “error bearing”) and the orientation of the semimajor axis of the error ellipse supplied by Argos (the “ellipse orientation”), the angles were plotted against one another for visual comparison and then a cross correlation analysis was performed between the two parameters for both processing methods and compared with the 95% confidence intervals assuming no correlation. Since the Argos ellipse orientation angle runs from 0 to 180°, not 360°, the error bearing was expressed as 0 to 180° by subtracting 180 from values greater than 180.

To determine if the Argos supplied error ellipse parameters given for location codes 1, 2, and 3 provide reasonable bounds on the true errors, we examined the percentages of our true locations that lay within the i) error radius R (the square root of the product of semimajor and semiminor axes of the error ellipse), ii) error ellipse, and iii) semimajor axis used as an error radius.

We also used our error data to calculate five different types of empirical error estimates using the data from location codes 3, 2, 1, 0, A, and B but not Z, both for LS and KF methods. In total the five error methods, six location codes and two processing types resulted in 60 different empirical error estimates. These error estimates were:

1 68% normal error circles with error radii calculated by assuming the errors were normally distributed and 68% of the 1-dimensional errors would be found between 0 and m+z_0.68_ · s, where m is the arithmetic mean, s the standard deviation, and z_0.68_ = 0.44.
2 68% lognormal error circles with error radii calculated assuming errors were lognormally distributed and 68 percent of the errors would lie between 0 and m* · (s*)^z^, where m* and s* are the geometric mean and multiplicative standard deviation and z equal to z_0.68_ (i.e., 0.44).
3 68^th^ percentile error circles with error radii calculated as the 68^th^ percentile value for the 1-dimensional error data.

For the next two methods we broke the errors down into latitude errors and longitude errors and computed error ellipses with east-west oriented semimajor and north-south oriented semiminor axes a and b rather than single error radii.

4 68% lognormal error ellipses with semimajor and semiminor axes of the error ellipses computed assuming the longitude errors and latitude errors were each individually lognormally distributed and estimating a (semimajor axis) as m_lon_* · (s_lon_*)^z^ and b (semiminor axis) as m_lat_* · (s_lat_*)^z^, where lat and lon indicate latitude and longitude values, respectively; z is z_0.82_; and 0.82 is the square root of 68% (calculated expressed as a decimal, or 68/100). Hence z_0.82_ = 0.7939.
5 68^th^ percentile error ellipses with semimajor axis a as the 82^nd^ percentile of distribution of longitude errors and semiminor b as the 82^nd^ percentile of the latitude errors. Again, the 82^nd^ percentile is the square root of the 68^th^ percentile, calculated from a decimal. So for the 68^th^ percentile, we calculated the 82^nd^ percentile for each axis.

To determine which of these empirical error estimates was most useful for future studies of ranging and habitat use, we ranked each estimate based on the total size of the circle or ellipse enclosed by the radius or semimajor/semiminor axes and by how close to the desired 68% the percent of locations were within each circle or ellipse. The ranking was created in the following way. Beginning with the LS data for each of the methods above, we computed the error circles or ellipses for each LC. The areas of the resulting figures and the percentage of points falling within each figure were calculated. The results were ranked separately according to enclosed area (smaller was better) and according to the absolute deviation from 68% (smaller was better). A composite score for each of the 30 combinations was calculated as the sum of the area ranking plus 20% of the deviation ranking. The 20% weight was used because being close to 68% was desirable but not nearly as desirable as having a small error circle or ellipse. The final score for each method was the average of the composite scores for all the LCs. This was repeated for the KF data.

## Results

### Argos Position Errors – Magnitude and Distribution

We tested the hypotheses that each of the fourteen 1-dimensional error distributions was well described by a normal distribution and by a lognormal distribution (Table 3). None of the 14 were well described by a normal distribution (Lilliefors test, P<0.001). In 13 of the 14 cases, the errors were well described by the lognormal distribution (Lilliefors test with alpha = 0.05). Only for Kalman LC 3 did the data differ significantly from the lognormal distribution (Lilliefors test, P = 0.021). The probability plot showed deviations from lognormality occurred only in the far extreme values that could be argued to have resulted from factors different from those that governed the majority of the error deviations. Due to the fact that over 90% of the distributions were fit by the lognormal, for the remainder of the paper we assume lognormal distributions for all LCs.

**Table 3:** Fits of position errors to normal and lognormal distributions.

	Least Squares		Kalman Filtering	
Location Code, LC	p values		p values	
	Normal	Lognormal	Normal	Lognormal
3	<0.001	0.090	<0.001	0.021
2	<0.001	0.117	<0.001	0.066
1	<0.001	>0.5	<0.001	0.068
0	<0.001	0.209	<0.001	0.239
A	<0.001	>0.5	<0.001	0.235
B	<0.001	0.153	<0.001	0.208
Z	<0.001	0.153	<0.001	0.208

Results for testing the hypotheses that the normal or lognormal distributions were reasonable fits to the observed location error data. (Lilliefors test with α = 0.05) The normal distribution was never a good fit to the data, while the lognormal usually was.

Overall, the error in the locations increased with location code as anticipated, with LC 3 the most accurate and LC Z the least accurate (Table 4). The most accurate locations (LC 3) had an average error of 400–500 m regardless of the processing method and assumed underlying distribution (Tables 4 and 5). Of interest is that error magnitudes for LC A locations were statistically indistinguishable from LC 1 for both KF and LS processing (Tukey multiple comparison test, 95% CI), although, measures of variability (s and s*) appeared somewhat larger for LC A. The magnitude of the errors for the two test units were not statistically significantly different for LS processing except for LC A (KS test, P = 0.048). For KF, processing location errors differed for LCs 1, A and B (KS tests, P<0.04 for all three). The errors for these 4 statistically significant differences averaged 60% larger for the transmitter constructed by North Star.

**Table 4:** Basic statistics for 1-dimensional location errors assuming data are normally distributed.

Comparison between Empirical Errors and Argos-provided errors, using the relationships in Table 2 for normally distributed data									
		Least Squares Data, N = 658				Kalman Filtered Data, N = 843			
Location Code, LC	Argos Quoted Error (“68^th^ percentile”) (m)	N	Mean (m)	Standard Deviation (m)	68^th^ percentile, empirical (m)	N	Mean (m)	Standard Deviation (m)	68^th^ percentile, empirical (m)
3	<250	94	482	493	478	131	507	413	512
2	250< <500	91	694	515	903	68	1088	1,200	1,220
1	500< <1500	78	1,881	2,192	1,764	78	1,932	1,532	2,485
0	>1500	44	4,941	3,472	5,620	41	4,833	3,789	5,019
A	No estimate (LS) or unbounded estimate (KF)	169	1,836	2,474	1,673	178	1827	2329	1701
B	No estimate (LS) or unbounded estimate (KF)	173	47,672	390,181	14,688	340	14,457	56,783	6,861
Z	Invalid location	9	1,257,087	3,015,949	26,341	7	16,538	21,140	14,235

These statistics are for the full dataset, not the location-matched pairs. Means and standard deviations were computed as in Table 2 for normally distributed data. The observed 68^th^ % confidence bound was calculated directly from the data. Argos–provided statistics are given for comparison. N is number of points. LS refers to least squares and KF to Kalman filter.

**Table 5:** Basic statistics for 1-dimensional location errors assuming the data are lognormally distributed.

Comparison between empirical errors and Argos-provided errors, using the relationships in Table 2 for lognormally distributed data											
		Least Squares Data, N = 658					Kalman Filtered Data, N = 843				
Location Code, LC	Argos Quoted Error (“68^th^ percentile”) (m)	N	Geometric mean, m* (m)	Multiplicative Standard Deviation, s*	68% Confidence Interval (m)	68^th^ Percentile (m)	N	Geometric Mean,m* (m)	Multiplicative Standard Deviation, s*	68% Confidence Interval (m)	68^th^ Percentile (m)
3	<250	94	367	2.15	167–772 (74%)	514 (73%)	131	373	2.32	161–867 (73%)	553 (72%)
2	250< <500	91	509	2.37	215–1,206 (70%)	762 (64%)	68	712	2.64	269–1,880 (75%)	1,121 (66%)
1	500< <1500	78	1,250	2.50	499–3,129 (72%)	1,920 (72%)	78	1,424	2.26	630–3,221 (63%)	2,086 (65%)
0	>1500 m	44	3,925	2.05	1,914–8,051 (73%)	5,493 (68%)	41	3,473	2.42	1435–8,406 (66%)	5,251 (71%)
A	No estimate (LS) or unbounded estimate (KF)	169	933	3.34	279–3,118 (68%)	1,640 (67%)	178	954	3.19	301–3035 (70%)	1,640 (69%)
B	No estimate (LS) or unbounded estimate (KF)	173	6,938	4.55	1,523–31,597 (73%)	14,098 (67%)	340	3,759	4.27	881–16,036 (71%)	7,409 (71%)
Z	Invalid location	9	29,862	19.56	1,527–583,996 (78%)	119,966 (78%)	7	8,868	3.46	2,565–30,656 (50%)	15,840 (67%)

These statistics are for the full dataset, not the location-matched pairs. Argos–provided statistics are given for comparison. 68% confidence intervals and 68^th^ percentile columns were computed using the relationships in Table 2. These percentiles may be compared with the empirical percentiles column in Table 4. The actual percentages for the 68% confidence intervals and 68^th^ percentiles were calculated from the data and are given in parentheses. N is the number of points. LS refers to least squares and KF to Kalman filter.

### LS versus KF Processing

Kalman filtering increased the total number of positions by 28% over least squares processing (Table 4). However, the increase in “good” positions (LC 1, 2, and 3) was only about 2% (263 LS and 277 KF). The increase in LC A positions was also modest, from 169 to 179 or 6%. The major increase in number of positions was in LC B positions which increased by 97% (173 LS to 340 KF).

In all, 16% of 658 matched-location pairs improved in location code and only 4% dropped a location code with KF processing. In addition, 168 more LC B positions became available that could not be computed by LS processing as KF processing allows positions to be computed from only 1 message received by the satellite.

Reprocessing with KF did not significantly improve location accuracy except for LC B positions. Mean error for LC 2 was significantly less for LS processing compared to KF (t = 2.25, DF = 134, P = 0.026). Mean error for LC B was significantly greater for LS processing compared to KF (t = 4.39, DF = 332, P<0.001). For the other location codes mean errors did not differ significantly (t <1.1, P>0.3).

### Argos Distance Errors - Bearings

**Figure 1:**
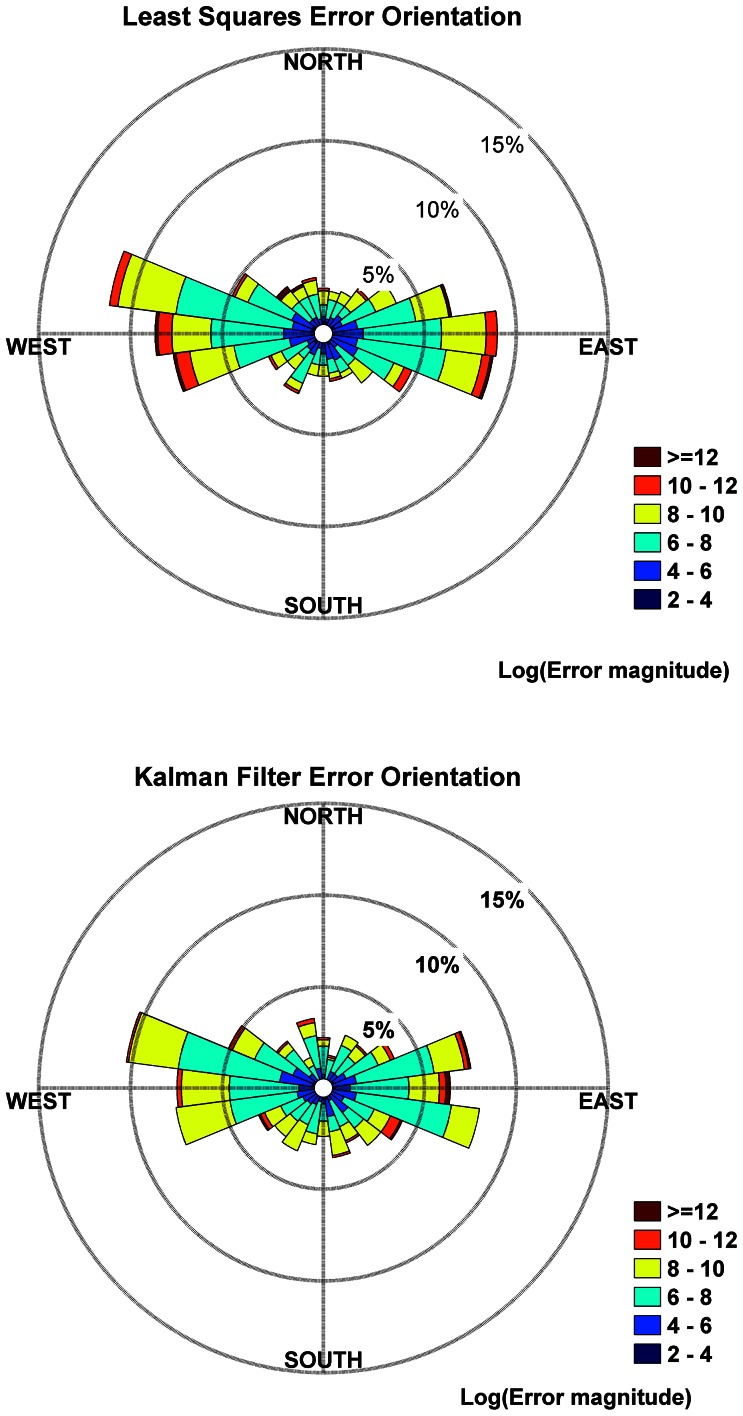
Radial histograms of the error orientations. Histograms are for all location codes combined; histograms are somewhat different for different location codes but give the same qualitative results. E-W errors predominate over and tend to be larger than N-S errors for both processing methods.

Longitudinal errors were larger than latitudinal error (Figure 1), with the difference being a function of the location code (Table 6). The ratio of the geometric means of longitude error and latitude error which quantifies the eccentricity of the error ellipses ranged from 2.3 to 2.8 for the best location codes (LC 3, 2, 1, and A). The ratio for LC 0 was 3.3, indicating strongly elliptical errors, while for LC B it was 1.3, indicating more circular errors (Table 6).

**Table 6:** Ratio of geometric mean longitude error to geometric mean latitude error.

Geometric mean longitude error/Geometric mean latitude error		
Location Code	Least Squares	Kalman Filtering
All	2.281	1.967
3	3.058	2.654
2	2.251	2.598
1	2.693	2.276
0	3.122	3.289
A	2.374	2.750
B	1.541	1.277

Any value of the ratio over 1 indicates the mean East-West error is greater than the mean North-South error.

A tendency for the Argos error ellipse orientation (direction of semimajor axis) to be east-west was also noted for both processing methods. However, visual examination of the plot of LS error ellipse orientations versus actual error bearings showed very little evidence of a correlation, which was confirmed with a cross correlation analysis. The cross correlation coefficient at 0 lag for LS data was −0.02, with 95% confidence interval ±0.08. For KF data the plot showed a hint of a correlation, and the cross correlation with all LCs combined (except Z) at 0 lag was significant at +0.14 (95% CI of ±0.07). This was found to be due to significant correlations at LC 3 (+0.19 with CI ±0.17) and LC B (+0.28 with CI ±0.11) but not at other location codes.

### Utility of Argos Error Estimates (1 and 2 dimensional)

Sixty-eighth percentile errors computed from the datasets were all larger than predicted by Argos for both KF and LS processing methods regardless of whether we assumed a normal (Table 4) or lognormal error distribution (Table 5). Under either error distribution assumption (normal or lognormal), the Argos given errors for LCs 1, 2, and 3 were smaller than either the empirically observed errors or the 68^th^ percentile estimated errors assuming lognormal distributions.

The percentage of true locations lying within the Argos error ellipse for LC 1, 2, and 3 positions was <25% for both processing methods (Table 7). The percentage was higher for location codes 0 and B (52% for LS; 61% and 54% for KF). Results were slightly higher for the percentages lying within the Argos error radius R: 17%–36% for LS processing and 14%–55% with KF processing. Percentages were highest for error circles of radius equal to the Argos semimajor ellipse axis: 46%–86% for LS processing and 38%–93% with KF processing.

**Table 7:** Percentage of true locations captured within error ellipse or circle.

LC	% within Argos Error Ellipse		% within Circle with Radius of Argos “Error Radius”		% within Circle of Radius Argos Semimajor Axis	
	Least Squares	Kalman Filter	Least Squares	Kalman Filter	Least Squares	Kalman Filter
3	25% (94)	15% (131)	17% (94)	14% (131)	46% (94)	38% (131)
2	24% (91)	19% (68)	29% (91)	15% (168)	69% (91)	52% (68)
1	22% (78)	24% (78)	28% (78)	21% (178)	77% (68)	74% (78)
0	52% (44)	61% (41)	36% (44)	34% (41)	86% (44)	93% (41)
A	N/A	29% (178)	N/A	35% (178)	N/A	74% (178)
B	N/A	54% (332)	N/A	55% (332)	N/A	86% (332)
Z	N/A	33% (6)	N/A	33% (6)	N/A	50% (6)

Percentages indicate the number of instances when the true position lay within the error ellipse or circle for that location code (LC). Numbers in parentheses are the total number of positions for the location code.

### Empirical Estimates of Accuracy

The rankings of the methods for computing the 68% empirical error ellipses were broadly similar for both LS and KF processing and whether all six LCs (LC 3 to LC B) were used or if only good LCs (LC 3, 2, 1, A) were considered. The best was the 68^th^ percentile error ellipses (technique #5). Second was 68^th^ percentile error circles from data (technique #3). The next best techniques were the 68% lognormal error ellipses (#4) and the 68% lognormal error circles (#2). By far the worst was the 68% normal error circles (#1). The semimajor (E-W) and semiminor (N-S) axes for the 5 techniques are presented in Table 8 for LCs 3, 2, 1, and A.

**Table 8:** Empirical estimates of the 68% accuracy ellipses/circles for LCs 3, 2, 1, and A.

LC	Method	Least Squares Positions		Kalman Filter Positions	
		E-W axis	N-S axis	E-W axis	N-S axis
3	1	0.713	0.713	0.700	0.700
	2	0.514	0.514	0.553	0.553
	3	0.478	0.478	0.512	0.512
	4	0.741	0.277	0.813	0.296
	**5**	**0.606**	**0.267**	**0.786**	**0.297**
2	1	0.935	0.935	1.649	1.649
	2	0.762	0.762	1.121	1.121
	3	0.903	0.903	1.220	1.220
	4	1.055	0.509	1.593	0.712
	**5**	**1.071**	**0.444**	**1.423**	**0.686**
1	1	2.906	2.906	2.648	2.648
	2	1.920	1.920	2.086	2.086
	3	1.764	1.764	2.485	2.485
	4	2.610	2.610	3.018	1.226
	**5**	**2.631**	**1.182**	**2.867**	**1.131**
A	1	2.993	2.993	2.916	2.916
	2	1.640	1.640	1.640	1.640
	3	1.673	1.673	1.701	1.701
	4	2.581	1.188	2.445	1.075
	**5**	**2.703**	**1.223**	**2.507**	**1.119**

See text for description of “Methods” column. For the best method, method 5 (boldface), the semimajor axis a was calculated as the 82^nd^ percentile of distribution of longitude errors and semiminor axis b was calculated as the 82^nd^ percentile of the latitude errors, meaning the ellipse so defined encloses approximately 68% of the true positions (note √0.68 is 0.82).

These results further demonstrate the importance of recognizing the lognormal nature of the error distributions. The 68^th^ percentile data circles (#3) by definition contained 68% of the locations, but technique #3 is not a viable option for estimating 2-dimensional error bounds for data points whose true location is unknown. (The other techniques contained from 61% to 94% of the total locations, which we considered acceptable.) Assuming data were normally distributed (#1) was by far the worst way to compute error circles/ellipses. The other three techniques (#2, #4, #5) assumed the data were lognormally distributed and they all performed quite similarly and quite well. The 68% lognormal error ellipse (#5) provides the best combination of small size and percent of locations included. This holds for both LS and KF locations and regardless of whether all location codes are included in the calculations or if just good locations are included (LCs 3, 2, 1, and A). For the three lognormal techniques (#2, #4, #5) the areas of the error ellipses for LS and KF locations were similar for all location codes except LC B and average areas were as follows: LC 3 = 0.74 km^2^, LC 2 = 2.6 km^2^, LC 1 = 11.2 km^2^, LC 0 = 70 km^2^, and LC A 9.0 km^2^. The error ellipses for LC B locations for LS and KF processing were 682 and 210 km^2^ respectively.

## Discussion

Relative location code accuracy varied for the most part as predicted, with location code 3 positions more accurate than LC 2, which were more accurate than LC 1 positions. Interestingly the LC A positions for which Argos provides no error estimates were found to be as accurate as the LC 1 positions, although LC A positions tend to have more outliers. This phenomenon has only been mentioned in passing in the literature (e.g., [23]), yet it has great potential importance. LC A is assigned to positions computed when only three messages are received at the satellite, and LC A positions are only available if users specifically subscribe to Service Plus/Auxiliary Location Processing [14]. Researchers who use only relatively high quality locations for their analyses (LCs 1, 2, and 3) could add perhaps 60% additional data to their data sets if they include LC A locations in their analyses, although they would need to be able to identify and remove large outliers. We suggest Argos publicize the likely value of LC A positions and encourage more users to request Service Plus/Auxiliary Location Processing or, preferably, supply users with positions for all location codes unless they specifically opt out.

We reject the hypothesis that Argos-provided estimates of error characterize the actual accuracy of positions. Error estimates provided by Argos were overly optimistic for both least squares processing and Kalman filtering. The issue is further complicated by the fact that Argos quotes its errors as “68^th^ percentile” yet lists ranges which appear more like confidence intervals and does not specify if the errors refer to position precision or position accuracy. Regardless, the Argos error estimates were too small whether they were considered confidence intervals, percentiles, or simply means of precision estimates or accuracy estimates. As a result, researchers cannot assume, as claimed by Argos, that 68% of locations fall within the ranges provided in Table 1. As noted above, multiple papers have reported the inadequacy of Argos-supplied error estimates to characterize position accuracies since at least the late 1990s but the values quoted by CLS, the Argos system operator, have remained unchanged.

We did accept the hypothesis that position errors – both 1-dimensional and 2-dimensional – were well-fit by the lognormal distribution and not by a normal – Gaussian – distribution. As mentioned above, this finding has been noted in several recent papers, but the significance has not been fully appreciated. The lognormal distribution is a skewed distribution, meaning large “outliers” are to be expected and are legitimate. The appropriate measure of central tendency is the geometric mean, not the arithmetic mean. Any statistical tests that assume a normal distribution, including t-tests, confidence intervals, and estimates of percentiles, must be performed on log transformed data if the results of the calculations are to be useful. Limpert et al. [31] review the properties of lognormal distributions and show they are widespread in the biological and physical world, in medicine, in economics, in linguistics, in social science, and in many other fields. They point out that only additive contributions to variability lead to normal distributions, and the much more commonly encountered multiplicative effects lead to the lognormal distribution. These authors found that only datasets composed of differences, sums, means, or other functions of original measurements were better fit by normal instead of lognormal distributions, suggesting that lognormal distributions may be much more common than frequently assumed.

Assuming lognormal distributions for the errors in position and circular error radii, our data report the errors in 68% of the LS positions can be expected to be less than 514 m for LC 3; 762 m for LC 2; 1,920 m for LC 1; 1,640 m for LC A; 5,493 m for LC 0; and 14,098 m for LC B. For KF locations, the errors in 68% of the positions can be expected to be less than 553 m for LC 3; 1,121 m for LC2; 2,086 m for LC 1; 1,640 m for LC A; 5,251 m for LC 0; and 7,409 m for LC B. (Table 5). In brief, “good” positions (LC 3, 2, 1, A) are accurate to about 2 km, LC 0 and B are accurate to about 5–10 km, but larger outliers are to be expected in all location codes and need to be accounted for in the user’s data processing.

Argos introduced KF processing with the hope that it would provide more and higher accuracy positions “especially for applications where just a few messages are received per satellite pass or for platforms operating in difficult transmission conditions” [14]. However our data show that the present KF formulation did not provide the expected across the board increase in accuracy under our conditions. In fact, for most location codes there was a tendency for LS errors to be somewhat smaller (Table 5). There were trivial increases in the number of locations of each location code generated with KF except with regards to LC B locations: there were nearly twice as many LC B positions for KF versus LS processing. The only meaningful advantage of KF processing that we observed is the ability to create fairly good LC B locations when only one message is received by the satellite, locations that cannot be computed using LS processing. Our results suggest that KF processing may exacerbate the difference in accuracy among different PTTs. This does not suggest that Kalman filtering should be discarded, only that the present formulation of the filter is not much of an improvement over LS for relatively benign transmission conditions. We suggest Argos examine different formulations of Kalman filters or look beyond to methods such as particle filters (e.g., [32]) if they wish to improve their positions for users under all conditions. They also need to incorporate the lognormal nature of the errors and to account for other sources of error that are not presently captured in the covariance matrix.

Previous studies [e.g., 19,22,23] have noted that east-west (longitudinal) error components were larger than north-south (latitudinal) components, which is to be expected because of the polar orbits of the satellites. We also observed this. However, our results suggest the axis of greatest error is tilted slightly north-south (Fig. 1). For the best location codes (3, 2, 1, A), the ratio of geometric mean east-west error is somewhat greater than 2.5 times that of the north-south error (Table 6), although it may vary somewhat with location code.

Argos provides error ellipses around many (LS) or all (KF) positions to account for this spatial nonuniformity in the error by computing semimajor and semiminor axes and orientation of the ellipse from the error covariance matrix and assumptions of normally distributed errors [15]. We anticipated a relationship between these error ellipses and the location errors calculated from the data. However, we found virtually no relationship between the orientations of Argos error ellipses and the actual bearings of the spatial errors. Nor do the magnitudes of the semimajor or semiminor axes correspond to the magnitudes of the actual spatial errors. Hence, for both LS and KF we must reject the hypothesis that the Argos error ellipses are useful for characterizing the accuracy of the calculated positions. We must also recommend against using these ellipses in studies of animal movement and habitat use. The Argos error ellipse included the true position only 15–29% of the time for location codes 3, 2, 1, and A (Table 7). The “error radius” (√a·b) was actually somewhat better (14–35%) and using the semimajor axis as an error radius performed best (38–78% ) (Table 7). We were surprised that the error ellipses failed so completely as estimates of true error. Do these error ellipses refer to the precision of the calculation rather than the accuracy of the result? Does the fact that Argos assumes in their calculations that the errors follow a normal distribution [15] have a major impact? Are there additional sources of errors that are not included in the error covariance matrix? We suggest Argos provide a better explanation of what their error ellipses describe and how they might be useful to users.

The empirically derived error circles and ellipses we calculated (Table 8) varied both in size and percent of locations included. In all cases they were much larger than the Argos provided error estimates. Due to the east-west bias of the errors inherent in Argos-generated locations, the calculated error ellipses were smaller in the north-south dimension and averaged smaller than our calculated error circles but contained similarly high proportions of the total locations. As a result, we feel that the ellipse semimajor and semiminor axis estimates provided in Table 8 are the most usable error estimates for future analysis of ranging and landscape usage.

### Conclusions

Our findings that actual position errors are larger than Argos-quoted errors are not new and broadly agree with what other researchers have found (see Table 4 summarizing others’ findings in [19]). What is new is our emphasis on the significance of the fact that Argos position errors are much better described by the lognormal rather than the normal distribution. The lognormal distribution is a skewed distribution, meaning large “outliers” are to be expected and are legitimate. Many of the commonly used methods of statistical analysis require an underlying normal distribution to be valid and thus give incorrect results when applied to untransformed lognormal data. Argos needs to redo their estimates of errors using the lognormal distribution in place of the normal distribution (or do their calculations on log transformed data). In addition, when supplying error ellipses based upon a proper formulation of the error covariance matrix, they should emphasize that many important factors contributing to position error are left out of their calculation and thus they significantly underestimate errors. It would be helpful if Argos could do their own research to give users some idea of the magnitude of the underestimation or improve them to truly include 68% of the locations.

Another important finding from this work is that as presently formulated, Kalman filtering does not improve the number or accuracy of any positions except those assigned location code B. For users who can utilize positions with an average error of up to 5–10 km (e.g., marine mammal studies and oceanographic drifters), Kalman filtering is a major improvement because of the large number of LC B positions provided. For users who can use positions with an average error of up to 2 km, either least squares or Kalman filtering works equally well. And for users who need most positional accuracies to be less than 1 km, Argos is not likely the correct technology for the project.

Our results found that LC A positions with no Argos assigned error have been underappreciated. They are equivalent in accuracy to location code 1 positions, although the variation in the data is greater. We feel that users should not have to go out of their way to request LC A positions as is presently the case.

While we acknowledge that differences among individual units can influence error estimates, the findings in this paper should be broadly applicable to a wide array of other PTTs used in tropical South America and most other geographical locations, regardless of how the units are packaged. It is known that broadband noise sources at times disrupt reception of Argos messages in southern Europe [21] and Central Asia [20], [33], but no such noise source has been reported in South America and we do not feel we observed any evidence of such. Other factors contributing to errors in position accuracies are not expected to depend upon the particular packaging design of our instrumentation and designs with similar components have been used in the past on non-psittacines with no reported issues. Since we used low power transmitters (0.25 W versus the frequently used 0.5 W and 1.0 W transmitters) and the environmental conditions under which we collected our data at fixed sites were more stable than they would be on a moving bird, we feel our results should give conservative estimates of true Argos errors in South America and other reasonably radio-quiet geographic regions.

We suggest it would be useful for other users to conduct experiments with fixed-site Argos PTTs of similar wattage in different locations of the world to verify that our findings are indeed globally applicable. Since temperature variations can theoretically impact the stability of the oscillator and electronics that generates the frequency of the PTT signal, tests under different temperature regimes would be particularly valuable. Finally, we hope that CLS will stop ignoring the findings of their users that their presently promulgated one-dimensional and two-dimensional (i.e., error ellipse) parameters are inaccurate and misleading and provide their users with better estimates of the accuracy of the positions they provide.
